# Supplementary material for: Perceived ability to comply with national COVID-19 mitigation strategies and their impact on household finances, food security, and mental well-being of medical and pharmacy students in Liberia
Source: PLoS One. 2021 Jul 9;16(7):e0254446. doi: 10.1371/journal.pone.0254446 (PMC8270202; doi:10.1371/journal.pone.0254446)
Supplement: S1 Table — (DOCX) [file pone.0254446.s002.docx]

**S1 Table: Characteristics of Household and Additional persons living in household**

| **Characteristics**  **N=113** | **N (%)** |
| --- | --- |
| Household: Type of property |  |
| Multi-unit property | 27 (23.9) |
| Single-unit property | 39 (34.5) |
| Dormitory, group home, assisted living | 18 (15.9) |
| Other | 18 (15.9) |
| Don’t know/Missing | 11 (9.7) |
| Household has electricity |  |
| Yes | 95 (84.1) |
| No | 16 (14.4) |
| Missing | 2 (1.8) |
| Household has a radio/television |  |
| Yes | 98 (86.7) |
| No | 13 (11.5) |
| Missing | 2 (1.8) |
| Someone in household owns a cellphone |  |
| Yes | 110 (97.3) |
| No | 1 (0.9) |
| Missing | 2 (1.8) |
| Everyone living in household owns a face mask |  |
| True | 110 (97.3) |
| False | 1 (0.9) |
| Missing | 2 (1.8) |
| Household has a place for handwashing |  |
| Yes | 104 (92.0) |
| No | 7 (6.2) |
| Missing | 2 (1.8) |
| Not counting self, is anyone in household currently pregnant |  |
| Yes | 11 (9.7) |
| No | 100 (88.5) |
| Missing | 2 (1.8) |
| How worried are you about the health of your household members due to COVID-19 |  |
| Not worried | 18 (15.9) |
| Somewhat worried | 38 (33.6) |
| Very worried | 51 (45.1) |
| Missing | 6 (5.3) |
