# Supplementary material for: Perceived ability to comply with national COVID-19 mitigation strategies and their impact on household finances, food security, and mental well-being of medical and pharmacy students in Liberia
Source: PLoS One. 2021 Jul 9;16(7):e0254446. doi: 10.1371/journal.pone.0254446 (PMC8270202; doi:10.1371/journal.pone.0254446)
Supplement: S2 Table — (DOCX) [file pone.0254446.s003.docx]

**S2 Table: Compliance with COVID-19 mitigation recommendations**

| **Questions**  **N=113** | **N (%)** |
| --- | --- |
| Are you currently able to follow stay-at home recommendations |  |
| Yes | 31 (27.4) |
| No | 77 (68.1) |
| Missing | 5 (4.4) |
| In past two weeks, how frequently have you left your home for work |  |
| Once per day | 15 (13.3) |
| 3-4 times per week | 19 (16.8) |
| 1-2 times per week | 25 (22.1) |
| I have not left my home for work | 8 (7.1) |
| Not applicable/not employed | 41 (36.3) |
| Missing | 5 (4.4) |
| In past two weeks, how frequently have you left your home to purchase goods for the household |  |
| Once per day | 29 (25.7) |
| 3-4 times per week | 28 (24.8) |
| 1-2 times per week | 44 (38.9) |
| Not applicable | 7 (6.2) |
| Missing | 5 (4.4) |
| In past two weeks, how often did you wear a face mask when you left home for work or to purchase goods |  |
| Never | 3 (2.7) |
| Sometimes | 14 (12.4) |
| Often | 23 (20.4) |
| Every time | 68 (60.2) |
| Missing | 5 (4.4) |
| In the past two weeks how often did you feel you could practice good social distancing when you left home |  |
| Never | 5 (4.4) |
| Sometimes | 45 (39.8) |
| Often | 33 (29.2) |
| Every time | 25 (22.1) |
| Missing | 5 (4.4) |
| Since the pandemic began in February 2020, do you feel your ability to receive medical care for other significant health issues has been impacted? |  |
| Yes | 63 (55.8) |
| No | 24 (21.2) |
| Don’t know/not sure | 20 (17.7) |
| Missing | 6 (5.3) |
| Do you feel Liberia’s experience with dealing with Ebola has prepared it to appropriately respond to COVID-19? |  |
| No, not prepared | 36 (31.9) |
| Somewhat prepared | 66 (58.4) |
| Very prepared | 5 (4.4) |
| Missing | 6 (5.3) |
