# Supplementary material for: Perceived ability to comply with national COVID-19 mitigation strategies and their impact on household finances, food security, and mental well-being of medical and pharmacy students in Liberia
Source: PLoS One. 2021 Jul 9;16(7):e0254446. doi: 10.1371/journal.pone.0254446 (PMC8270202; doi:10.1371/journal.pone.0254446)
Supplement: S3 Table — (DOCX) [file pone.0254446.s004.docx]

**S3 Table: Financial and Food Security Concerns Resulting from the COVID-19 Pandemic**

| **Questions**  **N=113** | **N (%)** |
| --- | --- |
| In last month have you experienced personal loss of income because of COVID |  |
| Yes | 76 (67.3) |
| No | 14 (12.4) |
| Not applicable | 18 (15.9) |
| Missing | 5 (4.4) |
| If yes, which contributed to loss of income |  |
| Fired/laid off | 1 (0.9) |
| Given time off without pay | 4 (3.5) |
| Given time off with reduced pay | 3 (2.7) |
| My hours were reduced | 4 (3.5) |
| I could not work and care for child in household | 12 (10.6) |
| I felt I was high risk for COVID and did not want to leave household | 15 (13.3) |
| My place of employment was temporarily closed | 8 (7.1) |
| Other | 35 (31.0) |
| None of above/Missing | 31 (27.4) |
| For how long can your HH follow stay-at-home recommendations before you are financially impacted |  |
| We are already experiencing financial impacts | 54 (47.8) |
| 1-2 more weeks | 6 (5.3) |
| 2-4 more weeks | - |
| 1-2 more months | 2 (1.8) |
| 3-4 more months | 2 (1.8) |
| For as long as Liberia feels it is necessary | 15 (13.3) |
| Don’t know | 29 (25.7) |
| Missing | 5 (4.4) |
| How worried are you about your HHs financial situation due to COVID |  |
| Not worried | 15 (13.3) |
| Somewhat worried | 30 (26.5) |
| Very worried | 63 (55.8) |
| Missing | 5 (4.4) |
| Which best reflects HH food situation over the last 7 days |  |
| I have had no difficulties eating enough food (normal pattern) | 60 (53.1) |
| I ate less preferred foods | 14 (12.4) |
| I skipped meals or ate less than usual | 22 (19.5) |
| I have gone at least one full day without eating | 4 (3.5) |
| I increased my food intake | 7 (6.2) |
| Missing | 6 (5.3) |
| Does your household have enough food stocks |  |
| Yes, enough for less than one week | 17 (15.0) |
| Yes, enough for one week | 21 (18.6) |
| Yes, enough for 1-2 weeks | 11 (9.7) |
| Yes, enough for 1 month | 8 (7.1) |
| Yes, enough for more than 1 month | 12 (10.6) |
| No | 39 (34.5) |
| Missing | 5 (4.4) |
