# Supplementary material for: Perceived ability to comply with national COVID-19 mitigation strategies and their impact on household finances, food security, and mental well-being of medical and pharmacy students in Liberia
Source: PLoS One. 2021 Jul 9;16(7):e0254446. doi: 10.1371/journal.pone.0254446 (PMC8270202; doi:10.1371/journal.pone.0254446)
Supplement: S4 Table — C1: First component from the principal component analysis with mixed data; C2: Second component; C3: Third component. (DOCX) [file pone.0254446.s005.docx]

**S4 Table: Loadings from principal component analysis with mixed data, combining the following variables: age, number of people in house, marital status, health concerns, loss of income, and gender.**

|  | **PC1** | **PC2** | **PC3** |
| --- | --- | --- | --- |
| Age | 0.445 | 0.021 | 0.261 |
| Number of people living in household | 0.031 | 0.534 | 0.071 |
| Married/cohabitating | 0.584 | 0.030 | 0.000 |
| Health: Very Worried | 0.017 | 0.691 | 0.005 |
| Loss of Income: Yes | 0.416 | 0.027 | 0.007 |
| Gender: Male | 0.002 | 0.001 | 0.866 |

PC1: first component from the principal component analysis with mixed data

PC2: second component

PC3: third component
