# Supplementary material for: Perceived ability to comply with national COVID-19 mitigation strategies and their impact on household finances, food security, and mental well-being of medical and pharmacy students in Liberia
Source: PLoS One. 2021 Jul 9;16(7):e0254446. doi: 10.1371/journal.pone.0254446 (PMC8270202; doi:10.1371/journal.pone.0254446)
Supplement: S5 Table — C1: First component from the principal component analysis with mixed data; C2: Second component; C3: Third component. (DOCX) [file pone.0254446.s006.docx]

**S5 Table: Loadings from principal component analysis with mixed data, combining the following variables: age, number of people in house, marital status, loss of income, and electricity.**

|  | **PC1** | **PC2** | **PC3** |
| --- | --- | --- | --- |
| Age | 0.672 | 0.010 | 0.033 |
| Number of people living in household | 0.000 | 0.709 | 0.002 |
| Married/cohabitating | 0.470 | 0.198 | 0.000 |
| Loss of Income: Yes | 0.339 | 0.047 | 0.220 |
| Gender: Male | 0.181 | 0.182 | 0.175 |
| Electricity: Yes | 0.008 | 0.006 | 0.668 |

PC1: first component from the principal component analysis with mixed data

PC2: second component

PC3: third component
