# Supplementary material for: Perceived ability to comply with national COVID-19 mitigation strategies and their impact on household finances, food security, and mental well-being of medical and pharmacy students in Liberia
Source: PLoS One. 2021 Jul 9;16(7):e0254446. doi: 10.1371/journal.pone.0254446 (PMC8270202; doi:10.1371/journal.pone.0254446)
Supplement: S6 Table — Categorical variables presented as absolute value (%); continuous variables presented via medians and interquartile range. Reference values: Gender, female; marital status, single/never married; worried about health, not worried/somewhat worried; loss of income, no/not applicable; worried about finances, not worried/somewhat worried; p-value computed via chi-square tests for categorical variables and Mann-Whitney-Wilcoxon test for numerical variables. (DOCX) [file pone.0254446.s007.docx]

**S6 Table: Factors associated with a positive depression screen (PHQ-8 ≥10)**

|  | **Depression Score <10** | **Depression Score ≥10** |  |
| --- | --- | --- | --- |
|  | **N=83** | **N=20** | **p-value** |
| Age | 28 [26,32] | 30 [27,32] | 0.45 |
| Male | 50 (60%) | 13 (65%) | 0.89 |
| Married/Cohabitating | 20 (24%) | 6 (30%) | 0.80 |
| Number of people living in household | 7 [5,10] | 10 [7,14] | 0.03 |
| Health: Very worried | 35 (42%) | 14 (70%) | 0.05 |
| Loss of income: Yes | 60 (72%) | 14 (70%) | 1.00 |
| Worried about finances: Very worried | 45 (54%) | 17 (85%) | 0.02 |

Categorical variables presented as absolute value (%); continuous variables presented via medians and interquartile range. Reference values: gender, female; marital status, single/never married; worried about health, not worried/somewhat worried; loss of income, no/not applicable; worried about finances, not worried/somewhat worried; p-value computed via chi-square tests for categorical variables and Mann-Whitney-Wilcoxon test for numerical variables
