# Supplementary material for: Perceived ability to comply with national COVID-19 mitigation strategies and their impact on household finances, food security, and mental well-being of medical and pharmacy students in Liberia
Source: PLoS One. 2021 Jul 9;16(7):e0254446. doi: 10.1371/journal.pone.0254446 (PMC8270202; doi:10.1371/journal.pone.0254446)
Supplement: S1 File — (PDF) [file pone.0254446.s008.pdf]

Invitation and Consent

You are being asked to participate in a survey to explore your knowledge, attitudes, and perceptions about the COVID-19 pandemic and its impact on your household´s ability to comply with Liberia´s current control measures. Please note, we want you to respond about the household where you have decided to spend most of your time during the COVID-19 Stay-At-Home order.

Please complete the survey below.

Thank you!

First Name ______________________

Last name ______________________

Signature ______________________

Demographic Information

You are being asked to participate in a survey for the purpose of investigating yuor knowledge, attitudes, and perceptions about the COVID-19 pandemic and its impact on your household´s ability to comply with Liberia´s current control measures.

Please complete the survey below.

Thank you!

1. Gender	◯ Male<br>◯ Female
2. What is your age in years?	__________________
3. Date of Birth<br><br>[PLEASE CONFIRM YOU FILLED OUT MONTH, DAY AND YEAR CORRECTLY BEFORE ADVANCING]	__________________
4. Marital Status	◯ Single never married<br>◯ Married<br>◯ Cohabitating<br>◯ Divorced<br>◯ Widowed
5. Occupation	◯ Employed for wages<br>◯ Self Employed<br>◯ Domestic work in someone else´s home<br>◯ Homemaker<br>◯ Farming<br>◯ Unemployed<br>◯ Student<br>◯ Retired<br>◯ Other
6. County of residence during the COVID-19 Stay-at-home order	◯ Bong<br>◯ Bomi<br>◯ Grand Bassa<br>◯ Grand Gedeh<br>◯ Grand Cape Mount<br>◯ Ghapolu<br>◯ Grand Kru<br>◯ Lofa<br>◯ Maryland<br>◯ Montserrado<br>◯ Margibi<br>◯ Nimba<br>◯ Rivercess<br>◯ River Gee<br>◯ Sinoe
7. What is the highest educational level you completed?	◯ Highschool graduate<br>◯ College 1 year to 3 years (some college or technical school)<br>◯ College 4 years or more (Completed college)

Household Information

Please complete the survey below.

Thank you!

8. Number of people [COUNTING YOURSELF] living in the household where you are spending the majority of your time during the COVID-19 Stay-at-home order

____

9. Including yourself, how many members of your household are > 60 years old?

____

10. Number of children < age 5 years old living in your household?

____

11. Which of these property types best describes the household where you currently Ispend the majority of your time during the COVID-19 Stay-at-home order?

- ◯ A multi-unit property. This includes a condominium, co-op, or building with two more units.
- ◯ A single-unit property. This includes a detached home or townhouse.
- ◯ A dormitory, group home, assisted living, or nursing home
- ◯ Other
- ◯ Don't know / Not sure

12. Has a doctor, nurse, or other health professional ever told you that you had any of the following?

[PLEASE SELECT ALL THAT APPLY]

- ☐ A heart attack, also called a myocardial infarction
- ☐ Angina or coronary heart disease
- ☐ High blood pressure
- ☐ Type II diabetes
- ☐ Cancer
- ☐ Asthma
- ☐ Chronic obstructive pulmonary disease (COPD), emphysema, or chronic bronchitis
- ☐ Kidney disease
- ☐ HIV
- ☐ Tuberculosis
- ☐ Ebola survivor
- ☐ Lassa Fever
- ☐ Immunodeficiency
- ☐ None of the above

13. Not counting yourself, does anyone in your household have any of the following?

[PLEASE SELECT ALL THAT APPLY]

- ☐ A heart attack, also called a myocardial infarction
- ☐ Angina or coronary heart disease
- ☐ High blood pressure
- ☐ Type II diabetes
- ☐ Cancer
- ☐ Asthma
- ☐ Chronic obstructive pulmonary disease (COPD), emphysema, or chronic bronchitis
- ☐ Kidney disease
- ☐ HIV
- ☐ Tuberculosis
- ☐ Has previously had Ebola and survived
- ☐ Has previously had Lassa Fever
- ☐ Immunodeficiency
- ☐ No

14. Are you currently pregnant?

- ◯ Yes
- ◯ No
- ◯ Not applicable

15. Not counting yourself, is anyone in your household pregnant?	◯ Yes<br>◯ No
16. Does your household have electricity?	◯ Yes<br>◯ No
17. Does your household have a radio/television?	◯ Yes<br>◯ No
18. Does anyone in your household have a cell phone?	◯ Yes<br>◯ No
19. Do you own a face mask?	◯ Yes<br>◯ No
20. Everyone living in my household has their own face mask?	◯ True<br>◯ False
21. What is the main source for drinking water for members in your household?<br><br>[PICK JUST ONE ANSWER]	◯ TUBE WELL OR BOREHOLE<br>◯ DUG WELL-PROTECTED WELL<br>◯ DUG WELL-UNPROTECTED WELL<br>◯ WATER FROM SPRING-PROTECTED SPRING<br>◯ WATER FROM SPRING-UNPROTECTED SPRING<br>◯ RAINWATER<br>◯ TANKER TRUCK<br>◯ CART WITH SMALL TANK<br>◯ SURFACE WATER (RIVER/POND/STREAM/IRRIGATION CHANNEL)<br>◯ BOTTLED WATER<br>◯ PIPED WATER- PIPED INTO DWELLING<br>◯ PIPED WATER- PIPED TO YARD/PLOT<br>◯ PIPED WATER- PIPED TO NEIGHBOR<br>◯ PIPED WATER- PUBLIC TAP/STANDPIPE<br>◯ Other
22. What is the length of time IN MINUTES that it takes to reach this source of drinking water?<br><br>[IF YOUR WATER SOURCES IS PIPED INTO YOUR HOUSE OR YARD PLEASE ENTER "0"]	______________________________<br>(provide time in minutes)
23. Does your household have a place for handwashing?	◯ Yes<br>◯ No
24. Currently, what kind of toilet facility do you and members of your household use?<br><br>[PICK JUST ONE ANSWER]	◯ NO FACILITY/FIELD,<br>◯ IN-DOOR TOILET<br>◯ TRADITIONAL PIT TOILET<br>◯ VENTILATED IMPROVED PIT LATRINE<br>◯ NEIGHBOR'S TRADITIONAL PIT TOILET<br>◯ NEIGHBOR'S IMPROVED PIT LATRINE<br>◯ OTHER

Precautions and Impact

Please complete the survey below.

Thank you!

25. Are you currently able to follow the Stay-at Home order?	◯ Yes<br>◯ No
26. In the past TWO WEEKS, what best describes how frequently you have left your home for work?	◯ Once a day<br>◯ Three to four times per week<br>◯ One to two times per week<br>◯ I have not left my house in the past two weeks<br>◯ Not applicable, not employed
27. In the past TWO WEEKS, what best describes how frequently you or a member of your household has left your home to purchase goods for the household?	◯ Once a day<br>◯ Three to four times per week<br>◯ One to two times per week<br>◯ I have not left my house in the past two weeks<br>◯ Not applicable
28. In the past TWO WEEKS, how often did you wear a face mask when you left the home for work or to purchase goods?	◯ Never<br>◯ Sometimes<br>◯ Often<br>◯ Every time
29. In the past TWO WEEKS, how often did you feel you could practice good social distancing when you left your home?<br><br>[social distancing = staying six feet away from other persons]	◯ Never<br>◯ Sometimes<br>◯ Often<br>◯ Every time
30. In the past MONTH, have you experienced a personal loss of income as a result of COVID-19?	◯ Yes<br>◯ No<br>◯ Not Applicable
31. If yes experienced a personal loss of income: Which of the following contributed to your personal loss of income?<br><br>[CHECK ALL THAT APPLY]	☐ I was fired/laid off<br>☐ I was given time off without pay (not fired, but not working)<br>☐ I was given time off with reduced pay (employer provided benefits)<br>☐ My hours were reduced<br>☐ I could not work and care for a child in the household<br>☐ I felt I was at high risk for COVID-19 and did not want to leave the home<br>☐ My place of employment was temporarily closed<br>☐ Other
32. For how long do you think your household can follow the Stay-at Home orders before you are financially impacted?	◯ We are already experiencing financial difficulty<br>◯ One-two more weeks<br>◯ Two-four more weeks<br>◯ One-two more months<br>◯ Three-four more months<br>◯ For as long as Liberia feels it is necessary<br>◯ Don't know

33. How worried are you about your household´s financial situation as a result of COVID-19?	◯ Not worried<br>◯ Somewhat worried<br>◯ Very worried
34. How worried are you about the health of your household members due to COVID-19:	◯ Not worried<br>◯ Somewhat worried<br>◯ Very worried
35. Which statement best reflects your households food situation over THE LAST 7 DAYS	◯ I have had no diffidulties eating enough food (normal pattern)<br>◯ I ate less preferred foods<br>◯ I skipped meals or ate less than usual<br>◯ I have gone at least one full day without eating<br>◯ I increased my food intake
36. Does your household have enough food stocks?<br><br>[Please pick the best answer that describes your household]	◯ Yes enough stock for less than one week<br>◯ Yes enough stock for one week<br>◯ Yes enough stock for 1-2 weeks<br>◯ Yes enough stock for 1 month<br>◯ Yes enough stock for more than 1 month<br>◯ No

Knowledge of COVID-19

Please complete the survey below.

Thank you!

37. COVID-19 is a virus that is thought to have been transmitted to humans from bats?	◯ True<br>◯ False
38. COVID-19 is transmitted through air	◯ True<br>◯ False
39. COVID-19 is transmitted through physical contact	◯ True<br>◯ False
40. COVID-19 is transmitted by touching surfaces	◯ True<br>◯ False
41. COVID-19 is transmitted through exposure to someones stool/feces	◯ True<br>◯ False
42. Which of the following are symptoms of COVID-19 infection<br><br>[CHECK ALL THAT APPLY]	☐ Cough<br>☐ Shortness of breath<br>☐ Fever<br>☐ Headache<br>☐ Sore throat<br>☐ Loss of taste or smell<br>☐ discoloration, lesions on the toes<br>☐ Vomiting<br>☐ Diarrhea<br>☐ Unexplained bleeding or bruising<br>☐ Weight loss
43. How long can you be infected with COVID-19 and not have symptoms	◯ 0-2 days<br>◯ 3-14 days<br>◯ > 2 weeks<br>◯ > one month<br>◯ Don't know
44. You can transmit the virus to others if you have no symptoms	◯ True<br>◯ False
45. Supportive care is the current treatment for COVID-19 in Liberia	◯ True<br>◯ False
46. Covering your nose and mouth while coughing, can help in the prevention of COVID-19 transmission	◯ True<br>◯ False
47. Washing your hands with soap and water can help in the prevention of COVID-19 transmission	◯ True<br>◯ False
48. Wearing a mask protects you from being infected with COVID-19	◯ True<br>◯ False
49. Wearing a mask protects you from giving COVID-19 to others	◯ True<br>◯ False

Attitudes and Perceptions about COVID-19

Please complete the survey below.

Thank you!

50. Most people who are infected with COVID-19 will die	◯ True<br>◯ False
51. Most people who are infected with COVID-19 will recover	◯ True<br>◯ False
52. Even though children seem to have less severe symptoms when infected with COVID-19, they can be an important source of transmission to others	◯ True<br>◯ False
53. Since the COVID-19 outbreak began in February and March 2020, do you feel that your ability to receive medical care for other significant health issues has been impacted?	◯ Yes<br>◯ No<br>◯ Don't know / Not sure
54. Do you know whom to contact if you or family member gets exposed to someone who is infected with COVID-19?	◯ Yes<br>◯ No
55. If you think someone in your household has been infected with COVID-19 where will you first seek treatment?<br><br>[CHOOSE ONE BEST ANSWER]	◯ Won´t seek treatment<br>◯ Hospital<br>◯ Local Pharmacy<br>◯ Traditional Healer<br>◯ Family member<br>◯ Church<br>◯ Don't Know
56. If you or a family member have to go to the health facility for COVID-19 how confident are you that the health facility will be able to help you or your family member?	◯ Not confident at all<br>◯ Somewhat confident<br>◯ Very confident
57. Do you feel that Liberia´s experience with dealing with Ebola has made Liberia prepared to appropriately respond to COVID-19?	◯ No, not prepared<br>◯ Somewhat prepared<br>◯ Very prepared

Questions about your state of mind

Please complete the survey below.

Thank you!

58. In the last TWO WEEKS, how many days did you have little interest or little happiness in doing things?	◯ NO DAYS<br>◯ LESS THAN 1 WEEK<br>◯ 1 WEEK OR MORE<br>◯ ALMOST ALL DAYS<br>◯ DON'T KNOW<br>◯ PREFER NOT TO ANSWER
59. In the last TWO WEEKS, how many days did you feel down, depressed or without motivation?	◯ NO DAYS<br>◯ LESS THAN 1 WEEK<br>◯ 1 WEEK OR MORE<br>◯ ALMOST ALL DAYS<br>◯ DON'T KNOW<br>◯ PREFER NOT TO ANSWER
60. In the last TWO WEEKS, how many days did you have difficulty sleeping, staying asleep, or sleeping more than is customary?	◯ NO DAYS<br>◯ LESS THAN 1 WEEK<br>◯ 1 WEEK OR MORE<br>◯ ALMOST ALL DAYS<br>◯ DON'T KNOW<br>◯ PREFER NOT TO ANSWER
61. In the last TWO WEEKS, how many days did you feel tired or with little energy?	◯ NO DAYS<br>◯ LESS THAN 1 WEEK<br>◯ 1 WEEK OR MORE<br>◯ ALMOST ALL DAYS<br>◯ DON'T KNOW<br>◯ PREFER NOT TO ANSWER
62. In the last TWO WEEKS, how many days did you have lack of appetite or ate less?	◯ NO DAYS<br>◯ LESS THAN 1 WEEK<br>◯ 1 WEEK OR MORE<br>◯ ALMOST ALL DAYS<br>◯ DON'T KNOW<br>◯ PREFER NOT TO ANSWER
63. In the last TWO WEEKS, how many days did you feel bad about yourself, thought you were a failure, or that you let down your family or yourself?	◯ NO DAYS<br>◯ LESS THAN 1 WEEK<br>◯ 1 WEEK OR MORE<br>◯ ALMOST ALL DAYS<br>◯ DON'T KNOW<br>◯ PREFER NOT TO ANSWER
64. In the last TWO WEEKS, how many days did you have difficulty concentrating on things (such as reading a newspaper, watching, or listening to the radio)?	◯ NO DAYS<br>◯ LESS THAN 1 WEEK<br>◯ 1 WEEK OR MORE<br>◯ ALMOST ALL DAYS<br>◯ DON'T KNOW<br>◯ PREFER NOT TO ANSWER

65. In the last TWO WEEKS, how many days did you feel slow in your movements or in speaking; or the contrary, in which you felt agitated and you stayed walking from one side to another, more than is customary?	◯ NO DAYS<br>◯ LESS THAN 1 WEEK<br>◯ 1 WEEK OR MORE<br>◯ ALMOST ALL DAYS<br>◯ DON'T KNOW<br>◯ PREFER NOT TO ANSWER
66. How much do you feel COVID-19 has contributed to the way you feel currently?	◯ I felt this way before COVID-19 arrived<br>◯ COVID-19 has worsened the way I feel<br>◯ COVID-19 has improved the way I feel<br>◯ Unsure/Don´t know
