# Supplementary material for: Perceived ability to comply with national COVID-19 mitigation strategies and their impact on household finances, food security, and mental well-being of medical and pharmacy students in Liberia
Source: PLoS One. 2021 Jul 9;16(7):e0254446. doi: 10.1371/journal.pone.0254446 (PMC8270202; doi:10.1371/journal.pone.0254446)
Supplement: Attachment — Submitted filename: 08122 Response to Reviewer_final.docx [file pone.0254446.s009.docx]

June 14, 2021

*PLOS ONE*

Editorial Office

To the Editor:

We appreciate the time and energy the reviewers put into providing very detailed comments for improvement of our manuscript and in the editor’s patience in waiting for our responses. We have done our best to respond to each of their comments. Below you will find the reviewer’s or editor’s comments in bold and our responses in italics. Thank you.

**Please include additional information regarding the survey or questionnaire used in the study and ensure that you have provided sufficient details that others could replicate the analyses. For instance, if you developed a questionnaire as part of this study and it is not under a copyright more restrictive than CC-BY, please include a copy, in both the original language and English, as Supporting Information.  If the original language is written in non-Latin characters, for example Amharic, Chinese, or Korean, please use a file format that ensures these characters are visible.**

*Response: Thank you we have uploaded a copy of the survey and added S8. Study Questionnaire to the supporting information section.*

**Review Comments to the Author**
**Reviewer #1: At first, when I read the text, I was surprised that it was about surveying students, because that was not mentioned in the abstract. The authors should better present that already there. Because this is crucial for the interpretation of the results. The fact that, for example, compliance with mask wearing is high could be explained by educational inequality.**

*Response: We have altered the first sentence of the Abstract Methods to the following: “We conducted an email-based survey among current medical and pharmacy students of the University of Liberia College of Health Sciences between July and October 2020.”*

**The study aims to describe the ability of Liberian households to comply with national mitigation strategies and the impact the pandemic is having on their finances, food security, and individual well-being. Although the limitations of the study state that students are not representative of the population as a whole due to their average age, they do not state that students and their family housholds per se represent a bias, since not everyone has access to higher education. I know very little about the education system in Liberia, unfortunately, but I suspect that access to university is highly socially selective for children from wealthier families. The authors should elaborate a bit on this.**

*Response: Thank you for this observation. We have adjusted the language of the Limitations Paragraph in the Discussion by adding the following: “Further, by targeting only households in which a medical or pharmacy student resides, we are likely limiting the generalizability of our results to households that may be more socio-economically advantaged compared to the general population.”*

**The response rate is high, but couldn't a non-responder analysis have provided a bit more coverage? The student data are known to the university administration and are certainly available in anonymized form. It would have been possible to show whether the realized sample is selectively biased, e.g. with regard to age or gender distribution.**

*Response: We thank the referee for this question. This is indeed the greatest limitation of our findings. Unfortunately, we do not have direct access to University data, which could be used to check representativeness of our sample. We are aware that it is very unlikely that those 113 participants represent the entire student population, forming thus a biased sample. As discussed in the last paragraph of the Discussion, starting on line 309 of the new clean version of manuscript, our sample was self-selected and likely biased towards younger and wealthier groups. Our results, nonetheless, showed that even this selected group of students, which in theory have more resources to comply with stay-at-home measures and also less likely to be food insecure, were still strongly affected by the pandemic.*

**Because of the things that have been mentioned, the study does report very relevant and important results, but I think one should not overestimate the scope of the study. For one thing, it just (the case of Ebola) suggests why the case of Liberia is a good starting point to make statements about the impact of the COVID pandemic in African countries. But in the introduction, I think there is too much focus on continents that are structurally disadvantaged in the pandemic. Here, it might be better to elaborate a bit more clearly on what can be learned from this country study. It should also be refelected that the acquisition via email and the resulting selection of the study population entails major limitations in the distribution range. The statistics here suggest too great a degree of objectively generalizable facts.**

*Response: We have added the following sentence to the end of the Introduction “With these results we hope to more specifically elucidate the country specific impacts of COVID-19 mitigation strategies on every day Liberians. These results will hopefully aid Liberia’s policy makers in placing resources where they can provide the most help and provide guidance to other low-and middle-income country stakeholders that may be grappling with similar issues.”*

*We have additionally updated the Limitations Paragraph in the Discussion to address the reviewers several comments in this respect.*

**Reviewer #2: This paper addresses the very important, original, and timely question of what drives households’ ability to comply with COVID restrictions and the latter’s impact on food insecurity, mental health, and finances in Liberia. The authors address this question using data collected from a survey of medical/ pharmacy students in Liberia, and by calculating descriptive statistics and conducting logistic regressions. The authors find that face mask use is high (>80%) but the ability to comply with social distancing was relatively low and only 13% reported that they felt they could fully adhere to the stay-at-home orders. Additional analyses explore socio-demographic correlates of these outcomes as well as poor mental health and food insecurity.
The study presents the results of original research, the results of which do not appear to have been reported elsewhere. The article is generally presented in an intelligible fashion and is written in standard English, although there are some important areas requiring clarification. However, I have some concerns about the paper. The sample size is extremely small and the authors need to do more to discuss the generalizability of the data. Furthermore, some of the analyses (methods and results) are difficult to understand and the conclusions could be foreshadowed with greater discussion of the literature motivating the variables under study.
Specific comments:
1. Abstract- ‘introduction’ should introduce the goals/ objectives of the paper.**

*Response: We have added the following sentence to the Abstract Introduction: “The aim of this study was to 1) understand the impact of student’s household’s ability to comply with COVID-19 mitigation strategies, 2) identify predictors of mitigation strategy compliance, and 3) describe the impact of COVID-19 on household economics, food-security, and mental well-being.*

**2. Title and abstract should reflect the focus on students in the data – e.g. substitute ‘household’ for ‘students’ in both.**

*Response: We have adjusted the title to the following: “Perceived ability to comply with national COVID-19 mitigation strategies and their impact on household finances, food security, and mental well-being of medical and pharmacy students in Liberia*

**3. p.4-5 Should do much more to motivate the importance of the study population -why in particular would survey responses from this student population be of interest? I imagine the authors could develop an argument for studying this population (e.g. because of wider discussion about how youth are most adversely affected by *the pandemic, and/or importance of studying healthcare professionals), but this needs to be stated explicitly.***

*Response: We have added the following sentence to the Study Design and Participant Recruitment section: “We chose to utilize students during this unique time when they were home with their families as they represented a study population for which we had email addresses; were a population with a high likelihood of responding to an email survey request; and represented a mechanism through which it was felt we could get a quick snap shot of conditions in their family households to which they had returned.”*

**4. Related, the Introduction also needs to do more to justify the focus on the socio-demographic variables ultimately examined in the statistical analyses. Presumably the decision to focus on e.g. age, gender, and concern about household members’ health was motivated by some theoretical expectation in the literature. Please outline in the Introduction. This would greatly improve the reader’s interpretation of the results/ overall takeaways of the statistical analyses.**

*Response:* *The following sentence was added to the Statistical Analysis section of the Methods: “All variables used in the statistical analysis were selected* a priori*, based on our theoretical expectations. We did not use automatic selection procedures and variables were not selected based on p-values observed in univariable regression, as these could lead to unreliable inference.”*

**5. Food vulnerability and food insecurity are used interchangeably in the paper (e.g. on p.6). The authors should be consistent in the terminology used. I also encourage the authors to define the term where they use it – this is important given ambiguous meaning of this term and varying definitions used in the literature.
6. Coding of ‘food vulnerability’ needs more justification, and greater alignment with other studies. One common approach in the literature is to categorize scales such as that used in this paper into moderate and moderate/severe food insecurity, see e.g Barlow et al. 2020 (Reference also provides some discussion of the meaning of food insecurity and relevant references to additional literature that could be useful to cite here – see my point no.5 above).**

**Barlow, P., Loopstra, R., Tarasuk, V. and Reeves, A., 2020. Liberal trade policy and food insecurity across the income distribution: an observational analysis in 132 countries, 2014–17. The Lancet Global Health, 8(8), pp.e1090-e1097.**

*Response: Thank you. We have adjusted the language throughout the manuscript to consistently use the terminology of food security.*

*Unlike the Barlow paper referenced in the reviewer’s comment, which utilized multiple questions to base their categorization of food secure or not, we based our categorization on only two questions that do not lend themselves for quantifying into moderate or moderate/severe categories. However, we attempt to better contextualize this by adding the following sentence to the limitations paragraph in the Discussion “Next, we tried to explore and highlight household food security in our population based on two simple questions related to the quantity of existing food stocks as well as changes in one’s food consumption. Many examples exist of more nuanced strategies and questioning for determining food security at individual, household, and country levels. Our questioning provides only a glimpse into this issue for our respondents and should be followed-up with more in-depth study.”*

**7. p.7 “Due to our sample size…” presumably the authors mean ‘due to our small sample size’? Please clarify. Please also provide more detail about the issues this raises and why PCA to identify three covariates was deemed appropriate. This will be helpful for readers less familiar with this approach and to provide a clearer justification for the statistical approach ultimately taken.**

*Response: The sentence referred to has been changed to state “Due to our small sample size…..”. Thank you for pointing this out.*

*This limited sample size, in particular the small number of participants with the outcome of interest (e.g. 40 participants reported some food insecurity), restricted the number of variables that could be used for adjustment. Often, a minimum of 10 cases/events per variable is suggested for reliable inference, while other authors suggest that this ratio may be even higher, for example 15 cases/events per variable. For our setting, we could then include a maximum of 3-4 variables for adjustment to avoid overfitting the data. Principal component analysis is a dimensionality-reduction method, that creates new variables which are functions of all other variables in the dataset in such a way that minimizes the loss of information.*

*We agree with the reviewer that this part needed to be better explained, so we added the following sentence to the Statistical Analysis section of the Methods: “**Due to our small sample size, adjusting for all covariates may lead to overfit and unreliable inference. To restrict the number of parameters to be estimated, principal component analysis (PCA) for mixed (quantitative and qualitive) data were used, with varimax-rotation [15,16]. The first three components were used to summarize all variables and used as covariates in the logistic regression. With three covariates we guarantee at least 10 events per variable, providing reliable inference for the parameters of interest.”*

**8. With respect to the small sample size, I’d like to see the authors do much more to demonstrate whether or not this is of concern. For example, can the authors compare some of the socio-demographics of respondents in their data with socio-demographics of the wider student population based on other larger scale surveys?**

*Response: See response above*

**9. The small sample is the greatest weakness of the paper and whilst I appreciate that rapid surveys are also necessary to conduct timely analyses of important questions such as this, the authors need to do more in the Methods to explicitly address this issue. The authors could point to several advantages of a small sample – e.g. it permitted a timely analysis, it was the largest sample feasible within the resources provided, it can provide potentially useful exploratory insights, potentially other reasons?**

*Response: We appreciate the reviewer’s comments and have adjusted the text in the Statistical Analysis section to read as follows: “Due to our small sample size, adjusting for all covariates may lead to overfit and unreliable inference. To restrict the number of parameters to be estimated, principal component analysis (PCA) for mixed (quantitative and qualitive) data were used, with varimax-rotation [15,16]. The first three components were used to summarize all variables and used as covariates in the logistic regression. With three covariates we guarantee at least 10 events per variable, providing reliable inference for the parameters of interest. The COVID-19 pandemic is highly dynamic situation with rapid changes happening at a societal level and constantly changing measures, such that it was felt a study which could be performed quickly may still be useful to analyze the impact of those measures at that specific moment of the pandemic. While a larger sample would be preferable, it would demand more time such that the situational context of mitigation strategies and their impacts could be completely different at survey onset compared to survey end and complicating data analysis and interpretation of the results. This study, thus, provides a snapshot of COVID-19 and its implications in this specific group during a time where stay-at-home measures were enacted.”*

**10. With respect to the presentation of the results, this is generally ok. Table 4 is difficult to read and I would like to see some text in the left hand column explaining what C1-C3 are. This would greatly improve the readability of the table which is at present difficult to understand.**

*Response: We apologize that this presentation made interpretation harder. Since C1-C3 are components derived from the principal component analysis, they are a combination of factors, as explained in the note below Table 4. C1-C3 from face masks, social distancing and stay-at-home analysis differ from those obtained from the food security analysis, it is hard to present them all in a single table. In order to hopefully clarify the table, we added text explaining the components C1-C3, as we believe that having it only as a note below the table was indeed not clear.*

*The text now before table 4, on page 11, now reads: A multivariable logistic regression was used to explore factors associated with adherence to COVID-19 mitigation recommendations and food security (Table 4). In order to not overfit the model, we first ran a principal component analysis (PCA) with mixed data to reduce the number of parameters into combinations with the best possible correlation, followed by a multivariable logistic regression using the first three principal components (PC1, PC2 and PC3) as covariates. The loadings corresponding to PC1, PC2 and PC3 that were used in the analysis of compliance to face mask, social distancing and to stay-at-home measures, are presented in Table S4. The first principal component, PC1, was related to participants that were, on average, older and living with a partner; PC2 was related to participants that were sharing the house with several other people and were, on average, “very concerned” about the health of their household; while PC3 was related to men. Similar steps were taken to find risk factors associated to food security; a principal component regression analysis was performed, and the first three components were used as covariates. Their loadings are presented in Table S5, suggesting that PC1 was again related to participants that were, on average, older and living with a partner; PC2 was related to participants that were sharing the house with several other people; and PC3 was related to participants with electricity in their home.*

**11. In the Discussion, please begin with a summary of the key results. This would greatly improve the discussion by demonstrating how many of the interesting points raised here are linked to the specific results and takeaways of this particular paper.**

*Response: Thank you, we have rearranged the paragraphs of the Discussion to ensure it begins with a summary of our key results.*

Please do not hesitate to contact me if any further information is necessary or would be beneficial.

Best wishes,

Troy D. Moon, MD, MPH

Associate Professor of Pediatrics,

Division of Pediatric Infectious Diseases

Vanderbilt Institute for Global Health
